# Using Unlabeled Information of Embryo Siblings from the Same Cohort Cycle to Enhance In Vitro Fertilization Implantation Prediction

**DOI:** 10.1002/advs.202207711

**Published:** 2023-07-28

**Authors:** Noam Tzukerman, Oded Rotem, Maya Tsarfati Shapiro, Ron Maor, Marcos Meseguer, Daniella Gilboa, Daniel S. Seidman, Assaf Zaritsky

**Affiliations:** ^1^ Department of Software and Information Systems Engineering Ben‐Gurion University of the Negev Beer‐Sheva 84105 Israel; ^2^ Research Division AIVF Ltd. Tel Aviv 69271 Israel; ^3^ IVI Foundation Instituto de Investigación Sanitaria La Fe Valencia 46026 Spain; ^4^ Department of Reproductive Medicine IVIRMA Valencia 46015 Valencia Spain; ^5^ The Sackler Faculty of Medicine Tel‐Aviv University Tel‐Aviv 69978 Israel

**Keywords:** cohort sibling embryos, in vitro fertilization, machine learning, predicting embryo implantation potential, semi‐supervised learning

## Abstract

High‐content time‐lapse embryo imaging assessed by machine learning is revolutionizing the field of in vitro fertilization (IVF). However, the vast majority of IVF embryos are not transferred to the uterus, and these masses of embryos with unknown implantation outcomes are ignored in current efforts that aim to predict implantation. Here, whether, and to what extent the information encoded within “sibling” embryos from the same IVF cohort contributes to the performance of machine learning‐based implantation prediction is explored. First, it is shown that the implantation outcome is correlated with attributes derived from the cohort siblings. Second, it is demonstrated that this unlabeled data boosts implantation prediction performance. Third, the cohort properties driving embryo prediction, especially those that rescued erroneous predictions, are characterized. The results suggest that predictive models for embryo implantation can benefit from the overlooked, widely available unlabeled data of sibling embryos by reducing the inherent noise of the individual transferred embryo.

## Graphical abstract

In vitro fertilization (IVF) is used to study the effect of genotypic and environmental variation on phenotype. The widely available non‐transferred “sibling” embryos contribute to machine learning IVF implantation prediction suggesting that the uncertainty in the implantation potential can be reduced by the correlated sibling embryos. Incorporating sibling features in artificial intelligence‐driven embryo implantation prediction can have direct translational implications.

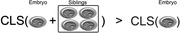

## 1 Introduction

Phenotypic variation is inherent in every biological system. A phenotype is determined by a combination of genetic and environmental factors. For example, the proportion of shared genetic background between human siblings can explain most of the variability in height,^[^ ^1^ ^]^ while environmental factors can affect gut microbiota composition,^[^ ^2^ ^]^ gene expression and disease susceptibility,^[^ ^3^ ^]^ and even lead to phenotypic variation in monozygotic (aka “identical”) twins.^[^ ^4^ ^]^ Thus, confinement of the genetic and environmental variability, for example, by considering siblings raised under similar conditions, can lead to reduced phenotypic variability, that is, increased phenotypic similarity. We hypothesized that such phenotypic correlations can provide predictive value regarding an individual's future phenotypic state by considering the phenotypic states of their siblings.

Specifically, during in vitro fertilization (IVF), a cohort of “sibling” oocytes, all sharing the same “parents” within the same IVF treatment, are fertilized and incubated for up to 6 days under the same laboratory conditions before one or a few embryos from the cohort are selected for transfer into the uterus. IVF embryo phenotypes are heavily affected by genetic^[^ ^5^ ^]^ and environmental^[^ ^6^ ^]^ factors, and thus the genetic and environmental variability is minimized for siblings from the same cohort.

Recent advances in time‐lapse video microscopy for live embryo imaging have transformed IVF into a data‐intensive field. This has led to innovative attempts to automatically and unbiasedly estimate the implantation potential of embryos based on an algorithmic assessment of their visual phenotypes and/or developmental trajectory.^[^ ^7^, ^8^, ^9^, ^10^, ^11^, ^12^ ^]^ Specifically, supervised machine learning has emerged as a powerful approach where features, computationally extracted from embryo images with known implantation outcomes, are used to train computational models to predict implantation.^[^ ^13^, ^14^, ^15^, ^16^, ^17^, ^18^, ^19^ ^]^ These models reach performance comparable to and even exceeding those of embryologists.^[^ ^20^, ^21^, ^22^ ^]^

However, from the available cohort, only one or at most two embryos are selected for transfer to the uterus. This poses a major limitation for machine learning‐based embryo selection approaches because the implantation potential of the majority of deselected embryos remains unknown.^[^ ^16^ ^]^ Thus, the number of embryos with known implantation outcomes available for training is severely limited. Recent studies have attempted to overcome this limitation by including “unlabeled” embryos in their model training schemes, specifically focusing on the subgroup of embryos found unsuitable for transfer to the uterus due to their poor morphological appearance and presumed limited implantation potential.^[^ ^23^, ^24^ ^]^ Other studies used the morphological annotations of embryos found unsuitable for transfer to the uterus to train models to predict the value of these morphological measurements as a readout for successful implantation potential.^[^ ^25^, ^26^ ^]^ However, none of these studies fully capitalized on and systematically assessed the potential of using the association between “sibling” embryos from the same cohort to enhance implantation prediction accuracy.

We asked whether we can take advantage of cohort‐derived information to train a machine‐learning model tasked with predicting implantation. We hypothesized that cohort embryos share information relevant to implantation prediction. Thus, using the full extent of the available unlabeled data in the cohort may provide further statistical discriminative power. Indeed, a few earlier studies provide evidence supporting the notion that cohort siblings encapsulate information that correlates with the transferred embryo's quality and implantation outcome such as cohort size,^[^ ^27^, ^28^, ^29^, ^30^, ^31^ ^]^ sibling blastocyst development,^[^ ^32^, ^34^ ^]^ or a combination of cohort‐specific variables.^[^ ^30^ ^]^ Here, we explicitly assess the contribution of sibling information to embryo implantation prediction by systematically evaluating different models trained with or without information from the cohort. We demonstrate that the unlabeled cohort embryos contribute to the prediction. Our results imply that artificial intelligence (AI)‐based embryo assessment can benefit from the widely available, and currently ignored, correlated data in the cohort's siblings. We characterize the specific properties of the cohort that contribute to the prediction and show that different cohort features can be exploited to enhance the performance of different models in varying contexts. Altogether, we suggest that considering the correlated properties of sibling embryos aids in canceling the inherently noisy single embryo prediction.

## 2 Results

### 2.1 Embryos from the Same Cohort are Phenotypically Correlated

**Figure 1:**
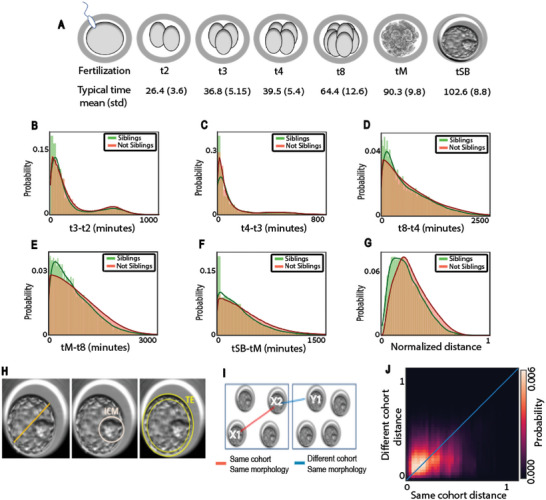
Sibling embryos from the same cohort are more similar than non‐siblings in terms of their morphokinetic properties. A) Schematic sketch. Morphokinetic features: cell division to the 2, 3, 4, and 8‐cell stage (t2, t3, t4, t8), the compaction of the morula – a day‐3 development stage (tM) and the start of blastulation (tSB) – a day‐5 development stage. B–G) Distribution of the difference in time intervals, in minutes (B–F) or normalized distance (G), between consecutive morphokinetic events compared across siblings versus non‐siblings embryo pairs. Curves were calculated with Kernel density estimation. *N* embryos = 16194. *N* cohorts = 1605. *N* positive cohorts = 928, *N* negative cohorts = 677. B) Mean (standard deviation) of distances between t3 and t2 intervals was 174.03 (214.7) for sibling embryos versus 202.26 (218.7) for non‐sibling embryos, Mann–Whitney‐U signed rank test *p*‐value < 0.0001. C) Mean (standard deviation) of distances between t4 and t3 intervals was 146.48 (220.56) for sibling embryos versus 158.75 (225.52) for non‐sibling embryos, Mann–Whitney‐U signed rank test *p*‐value < 0.0001. D) Mean (standard deviation) of distances between t8 and t4 intervals was 594.58 (531.66) for sibling embryos versus 684.09 (569.68) for non‐sibling embryos, Mann–Whitney‐U signed rank test *p*‐value < 0.0001. E) Mean (standard deviation) of distances between tM and t8 intervals was 691.03 (546.57) for sibling embryos versus 815.16 (602.9) for non‐sibling embryos, Mann–Whitney‐U signed rank test *p*‐value < 0.0001. F) Mean (standard deviation) of distances between tSB and tM intervals was 344.50 (336.89) for sibling embryos versus 434.79 (372.23) for non‐sibling embryos, Mann–Whitney‐U signed rank test *p*‐value < 0.0001. G) Mean (standard deviation) of normalized distances between all morphokinetic features time intervals was 0.241 (0.14) for sibling embryos versus 0.28 (0.15) for non‐sibling embryos, Mann–Whitney‐U signed rank test *p*‐value < 0.0001. H) Predefined criteria of blastocyst quality according to the Gardner three‐part scoring scheme. From left to right: Blastocyst expansion – volume and degree of expansion of the blastocyst cavity (ranked 1–6). Morphology of the Inner cell mass (ICM) –size and compaction of the mass of cells that eventually form the fetus (ranked A–D). Morphology of the trophectoderm (TE) – number and cohesiveness of the single cell layer on the outer edge of the blastocyst that eventually forms the placenta (ranked A–D). I) Schematic sketch of the analysis comparing embryo triplets: two from the same cohort (annotated X1 and X2), and two from a different cohort (X2 and Y1), where all embryos have the same Gardner annotations (similar morphological quality). J) Mean (standard deviation) of normalized distances between all morphokinetic features time intervals was 0.224 (0.141) for sibling embryos with similar Gardner scores versus 0.263 (0.146) for non‐sibling embryos with similar Gardner scores, Mann–Whitney‐U signed rank test *p*‐value < 0.0001. *N* = 1654732 ordered triplets.

Our data included information derived from 2089 transferred embryos collected from 1605 IVF cycles. These cycles included 1176 implanted blastocysts (positive embryos), 913 non‐implanted blastocysts (negative embryos), and 14 105 sibling embryos that were not transferred (Experimental Section, Table S1, Supporting Information). The timing of seven hallmark stages in embryo development, termed morphokinetic events, were manually annotated based on time‐lapse observation of the developing embryos (**Figure** **1**A, Table S1, Supporting Information, and Experimental Section). These morphokinetic events are considered key in proper embryo development and were shown to be correlated with implantation potential.^[^ ^10^, ^33^, ^34^, ^35^ ^]^ The timing of morphokinetic events from fertilization and the duration between consecutive morphokinetic events were more similar among sibling embryos than for randomly selected non‐sibling embryos, indicating lower intra‐cohort variability (Figure S1A–F, Supporting Information – timing of morphokinetic events, Figure 1B–F – time intervals between consecutive morphokinetic events, Figure 1G – normalized distances between multivariate representations of all time intervals between consecutive morphokinetic events, and Figure S1G, Supporting Information – normalized distances between multivariate representations of all morphokinetic events). Intriguingly, this deviation between intra‐ and inter‐cohort morphokinetic events duration increased at later stages of embryonic development (Figure S2, Supporting Information).

To verify that this higher intra‐cohort (i.e., between siblings) similarity is not an artifact due to the random selection of embryos, we compared intra‐ versus inter‐cohort similarity in the morphokinetic profiles of embryos with similar morphological qualities. We considered the manually annotated Gardner and Schoolcraft alphanumeric quality scoring scheme as a proxy for embryo morphological‐based quality, which is based on the assessment of three parameters: blastocyst expansion status, morphology of the inner cell mass (ICM), and morphology of the trophectoderm (TE)^[^ ^36^, ^37^ ^]^ (Figure 1H). We considered all possible ordered combinations of triplets of embryos that include a pair of sibling embryos from the same cohort and a third non‐sibling embryo from a different cohort, where all three embryos had the same Gardner scores (Figure 1I, Experimental Section). This analysis established that morphokinetic multivariate profiles of sibling embryos are more similar than non‐sibling embryos and that this similarity is not a mere consequence of a morphologic similarity between siblings at the blastocyst stage (Figure 1J). Altogether, this data supports the notion that sibling embryos share similar phenotypic properties.

### 2.2 Cohort Properties Correlate with Implantation Outcome

**Figure 2:**
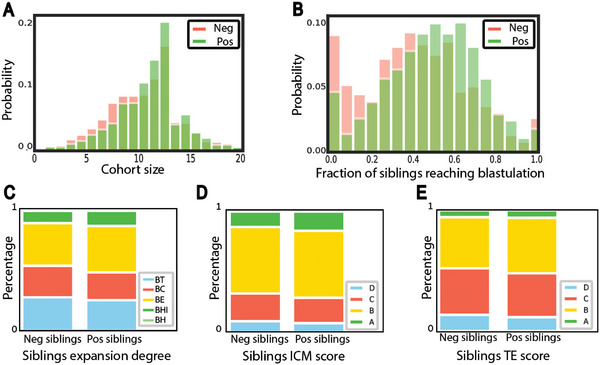
Siblings in positive cohorts are of higher morphological quality than those in negative cohorts. A,B) Distribution of the cohort size (i.e., number of sibling embryos in a cohort) (A) or the fraction of embryos within a cohort (not including the transferred embryo/s) to develop into a blastocyst (B) compared across positive versus negative cohorts. *N* transferred blastocysts = 2089. *N* implanted blastocyst = 1176, *N* non‐implanted blastocyst = 913. *N* cohorts = 1605. *N* positive cohorts = 928, *N* negative cohorts = 677. A) Mean (standard deviation) cohort size was 10.56 (3.19) for positive cohorts versus 9.9 (3.36) for negative cohorts, Mann–Whitney‐U signed rank test *p*‐value < 0.0001. B) Mean (standard deviation) fraction of sibling embryos within a cohort (not including the transferred embryo/s) reaching blastulation was 0.49 (0.22) for positive cohorts versus 0.41 (0.25) for negative cohorts, Mann–Whitney‐U signed rank test *p*‐value < 0.0001. C–E) Distribution of manually annotated Gardner scores of cohort embryos (not including the transferred embryo/s) across positive versus negative cohorts. *N* transferred blastocysts = 1936 embryos. *N* implanted blastocysts = 1141, *N* non‐implanted blastocysts = 795. C) Expansion degree: BT‐ early blastocyst, BC ‐ full blastocyst, BE‐ expanded blastocyst, Bhi ‐hatching blastocyst, BH ‐ hatched blastocyst. The total number of BH embryos in our dataset is three for implanted blastocysts and three for non‐implanted blastocysts, and thus cannot be seen in the graph. Corresponding Mann–Whitney‐U signed rank tests on the null hypothesis that the two schemes were drawn from the same matched distribution *p*‐value < 0.0001. D) Inner cell mass (ICM): ranked from A (high quality) to D (low quality). Corresponding Mann–Whitney‐U signed rank tests on the null hypothesis that the two schemes were drawn from the same matched distribution *p*‐value < 0.0001. E) Trophectoderm (TE): A (high quality) to D (low quality). Corresponding Mann–Whitney‐U signed rank tests on the null hypothesis that the two schemes were drawn from the same matched distribution *p*‐value < 0.0001.

The phenotypic similarity between siblings from the same cohort raises the hypothesis that morphological and morphokinetic properties of sibling embryos are correlated to the implantation outcome. To test this hypothesis, we compared the distribution of several cohort‐related properties for cohorts that included successfully implanted embryos (positive cohorts) versus those cohorts where the transferred embryo/s failed to implant (negative cohorts). First, we validated that positive cohorts contained more embryos than negative cohorts (**Figure** **2**A) and that the fraction of sibling embryos within a cohort (not including the transferred embryo/s) reaching blastulation was larger in positive cohorts (Figure 2B). These results are in agreement with previous reports for cohort size^[^ ^24^, ^25^, ^26^, ^27^, ^28^, ^29^, ^30^, ^31^, ^38^ ^]^ and sibling blastocyst development.^[^ ^32^, ^34^ ^]^ Each of the three Gardner morphological scores was elevated for sibling embryos, in positive cohorts compared to negative cohorts (Figure 2C–E). While the enrichments of higher‐quality cohort properties in positive cohorts were relatively small for each cohort property, they were all consistent toward favoring higher‐quality cohort properties in positive cohorts. Cumulatively, these results conclude that morphological properties of “sibling” embryos within a cohort, that were not transferred, are associated with the implantation potential of the embryo that was transferred within that cohort. These results establish that properties derived from sibling embryos correlate with the clinical outcome of their sibling‐transferred embryo.

### 2.3 Cohort Properties Contribute to Implantation Prediction

**Figure 3:**
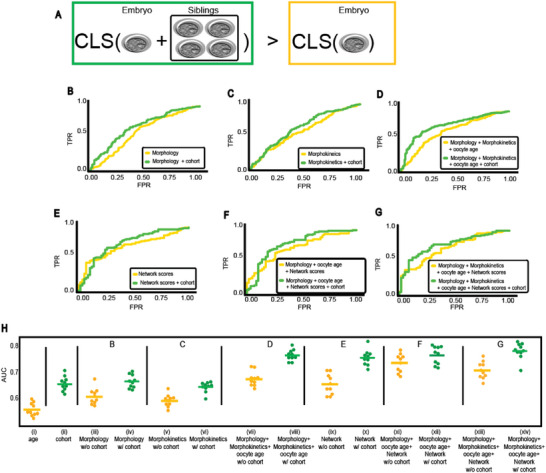
Implantation prediction performance (ROC‐AUC) comparison of different model pairs with versus without sibling features establishes that cohort properties contribute to implantation prediction. Statistical evaluation was computed with Wilcoxon signed rank test rejecting the null hypothesis that both models' predictions are drawn from the same distribution. A) Illustration: do cohort siblings data contribute to implantation prediction? B–G) Receiver operating characteristic (ROC) analysis and confusion matrices (Figure S4, Supporting Information) were calculated based on the test set. AUC increase of 0.04–0.1 from the inclusion of cohort features. B) Morphology (Gardner scores). *N* = 1936 (*N* test = 388) embryos. *N* positive embryos = 1141 (229), *N* negative embryos = 795 (159). AUC: 0.6 versus 0.68, respectively, *p*‐value < 0.0001. C) Morphokinetics. *N* = 2089 (*N* test = 418) embryos. *N* positive embryos = 1176 (235), *N* negative embryos = 913 (183). AUC: 0.591 and 0.641, respectively, *p*‐value < 0.0001. D) Morphokinetics, morphology, and oocyte age: *N* = 1936 (*N* test = 388) embryos. *N* positive embryos = 1141 (229), *N* negative embryos = 795 (159). AUC: 0.662 versus 0.764, respectively, *p*‐value < 0.0001. E–G) Deep convolutional neural network without (E) and with (F) morphology and oocyte age, and with morphokinetics, morphology, and oocyte age (G). *N* = 772 (*N* test = 155) embryos. *N* positive embryos = 482 (97), *N* negative embryos = 290 (58). E) Test AUC: 0.698 versus 0.744, respectively, *p*‐value < 0.001. F) Test AUC: 0.72 versus 0.779, respectively, *p*‐value < 0.001. G) AUC: 0.727 versus 0.8, respectively, *p*‐value < 0.001. H) Replication AUC analysis. Performance assessment for models trained without versus with cohort features in ten independent partitioning to train and test sets. Letters relate to the corresponding ROC panels (B–G). i) Age. AUC mean (standard deviation): 0.56 (0.02). ii) Cohort features. AUC mean (standard deviation): 0.65 (0.02) iii,iv) Morphology without (iii) or with (iv) cohort features. AUC mean (standard deviation) was 0.59 (0.02) without cohort versus 0.65 (0.02) with cohorts, Wilcoxon signed rank test *p*‐value < 0.01. v,vi) Morphokinetics without (v) or with (vi) cohort features. ACU mean (standard deviation) was 0.57 (0.02) without cohort versus 0.62 (0.01) with cohort, Wilcoxon signed rank test *p*‐value < 0.01. Surprisingly, the model trained with morphokinetic and cohort features performed slightly worse than the model trained with cohort features alone (ii vs vi) perhaps due to the inclusion of morphological features less correlative with the outcome without feature selection. vii,viii) Morphokinetics, morphology, and oocyte age without (vii) or with (viii) cohort features. AUC mean (standard deviation) was 0.65 (0.02) without cohort versus 0.74 (0.02) with cohort, Wilcoxon signed rank test *p*‐value < 0.01. ix,x) Deep convolutional neural network scores without (ix) or with (x) cohort features. AUC mean (standard deviation) 0.63 (0.03) without cohort versus 0.73 (0.02) with cohort, Wilcoxon signed rank test *p*‐value < 0.01. xi,xii) Deep convolutional neural network scores with morphology and oocyte age without (xi) or with (xii) cohort features. AUC mean (standard deviation) was 0.71 (0.03) without cohort versus 0.75 (0.03) with cohort, Wilcoxon signed rank test *p*‐value < 0.01. xiii,xiv) Deep convolutional neural network scores with morphokinetics, morphology, and oocyte age without (xiii) or with (xiv) cohort features. AUC mean (standard deviation) was 0.69 (0.03) without cohort versus 0.77 (0.02) with cohort, Wilcoxon signed rank test *p*‐value < 0.01.

Given that cohort properties were correlated to the implantation outcome, we hypothesized that the inclusion of cohort‐derived features can enhance the prediction power of a machine learning model initially trained without cohort information (**Figure** **3**A). To assess the generality of this idea, we trained several distinct machine learning models for the prediction of implantation outcome, each of these models was trained without and with cohort‐derived features. The performance of each pair of models, without or with cohort features, was compared to assess the contribution of the cohort information. The first model was morphology‐based, and trained on manually annotated Gardner scores (Experimental Section). The second model was morphokinetics‐based,^[^ ^7^ ^]^ and trained on manually annotated key morphokinetic events (Experimental Section). The third model combined morphology, morphokinetics, and the oocyte age, where the latter is widely accepted as correlated with implantation success^[^ ^39^, ^40^ ^]^ (Experimental Section). 17 cohort‐derived features were calculated from the siblings of the transferred embryo at the test. These included cohort size, fraction of sibling embryos reaching blastulation, and features encoding the siblings' Gardner scores (Experimental Section). The performance of each of these three models was improved by incorporating cohort‐derived features, as measured by the receiver operating characteristic (ROC) area under the curve (AUC) (Figure 3B–D).

Next, we turned to evaluate a deep convolutional neural network model that extracts information directly from the raw embryo images. These “deep learning” models were shown to surpass more traditional machine learning models in many domains, including IVF embryo implantation prediction.^[^ ^13^, ^14^, ^15^, ^16^ ^]^ Specifically, we used a pre‐trained VGG16 network^[^ ^41^ ^]^ and fine‐tuned it using preprocessed images of transferred blastocysts. The network's classifier score encodes the image‐based embryo's morphological information that is important for implantation prediction and was verified to be mildly more similar among transferred sibling embryos than for randomly selected non‐sibling transferred embryos (Figure S3, Supporting Information). Here too, we trained one model without cohort features, and another with the network's confidence score along with cohort features (Experimental Section). Similar to the previous models, the inclusion of cohort features enhanced the model's capacity to accurately predict implantation outcomes (Figure 3E). Moreover, cohort information enhanced the capacity to accurately predict implantation for a model that combined the deep learning model score, morphology features, and the oocyte age (Figure 3F), as well as for a model that also included morphokinetic features (Figure 3G). Finally, we validated that these results were consistent by performing tenfold cross‐validation: ten rounds of training and evaluation for each model, each time with an independent partitioning of cohorts to train and test sets (Figure 3H). Altogether, the inclusion of cohort‐derived features enhanced implantation prediction AUC by 0.04–0.1, where the best‐performing model had an AUC improvement from 0.73 to 0.80. These results, consistent across five models and multiple replicates, established that the siblings of the cohort encapsulate valuable information regarding the implantation potential of the transferred embryo.

### 2.4 Identifying Cohort Properties Driving the Model's Prediction

While cohort information was found to enhance implantation prediction, it was not clear which of the cohort features contributed to the measured boost in performance. Thus, we next aimed toward explaining the models' decisions. We focused our efforts on the two top‐performing models trained without or with the deep learning network's feature (i.e., confidence score), namely, a model trained on morphology, morphokinetics, and oocyte age. This enabled us, beyond plain model interpretability, to assess what information was encapsulated in the network beyond the morphology, morphokinetics, and oocyte age.

**Figure 4:**
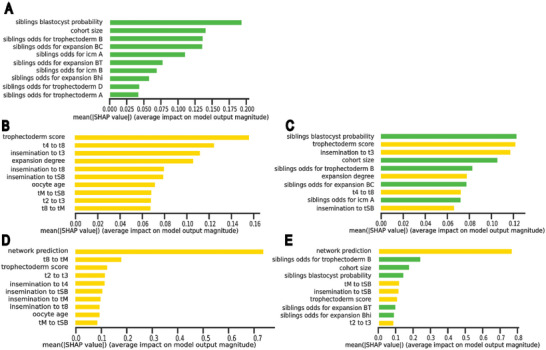
Model explainability analysis. Each panel shows features importance of the top ten features for a specific model using Shapely Additive Explanations (ShAP). A) Only cohort features. B,C) Morphokinetics, morphology, and oocyte age without (B) or with (C) cohort features. D,E) Deep convolutional neural network predictions with morphokinetics, morphology, oocyte age, and without (D) or with (E) cohort features.

To pinpoint what features were the most important for the model's prediction, we applied SHapley Additive exPlanations (SHAP), a game theory‐based method for interpreting models' predictions that assigns each feature an importance value for a prediction.^[^ ^42^ ^]^ For the model that was trained solely with cohort features, we found that the fraction of siblings reaching blastulation and the cohort size were the two most important features for implantation prediction (**Figure** **4**A). The trophectoderm score and expansion degree (morphology), oocyte age, and seven morphokinetic features were identified as the ten top features in a model trained with morphokinetics, morphology, and oocyte age (Figure 4B). When comparing the ten top‐ranked features with a model trained with cohort features, we noticed that the fraction of sibling embryos reaching blastulation came up as the most important feature for the prediction of implantation outcome, preceding morphology, morphokinetic and oocyte age (Figure 4C). Overall, five of the top ten important features were attributed to the cohort, and the other five were attributed to the transferred blastocyst (Figure 4C). The network confidence score was the most informative feature, by a margin, in a model trained with the network confidence score, morphology (Gardner scores), morphokinetics, and oocyte age (Figure 4D). The trophectoderm score was ranked only third and the expansion degree was not ranked within the ten most important features indicating that the network encoded the annotated morphology (Gardner scores). Cohort features were ranked higher than the morphology and morphokinetic features in a model that was trained with network prediction, morphology, morphokinetics, and oocyte age (Figure 4E). Half of the top features (5/10) were cohort‐related suggesting that the cohort encodes information that is not included within the embryo's image (Figure 4E). While oocyte age was identified as an important feature in the two models that did not include cohort features (Figure 4B,D), it was not one of the top ten features when cohort features were included (Figure 4C,E). This suggests that the cohort encodes information more discriminative than the oocyte age in terms of implantation prediction. Altogether, these analyses show that cohort features are an important source of information for the prediction of implantation outcomes.

### 2.5 Identifying Cohort Properties that Corrected Erroneous Prediction

**Figure 5:**
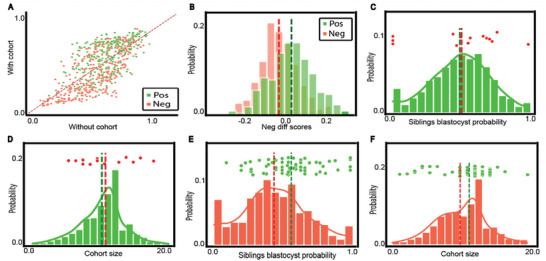
Analysis of cohort properties that “rescued” erroneous prediction. *N* transferred blastocysts = 2089 (*N* test = 388) from which 1176 (229) were positive and 913 (159) were negative embryos. The results refer to a model trained with morphology, morphokinetics, and oocyte age without and with cohort features. A) Embryos matched classification scores by the two models, without (*x*‐axis) and with (*y*‐axis) cohort features. B) Distribution of the difference in the embryos matched classification scores: with – without cohort features. Mean (standard deviation) difference for positive cohorts was 0.043 (0.1) (Wilcoxon signed rank test *p*‐value < 0.0001) versus −0.02 (0.09) (Wilcoxon rank‐sum test *p*‐value < 0.01) for negative cohorts. C–F) Distribution of fraction of blastocysts siblings (C,E) or cohort size (D,F) for positive (green, C,D) or negative (red, E,F) embryos. Each of the data points above the distribution indicate an embryo that was “rescued” with the cohort feature, that is, classified erroneously by a model trained without and corrected with a model trained with cohort features. C,D) Negative embryos that were erroneously classified as positive without cohort features and were correctly classified by a model that had access to cohort features. *N* = 15 rescued embryos. C) Mean (standard deviation) fraction of sibling embryos within a cohort (not including the transferred embryo/s) reaching blastulation was 0.49 (0.22) for positive cohorts versus 0.5 (0.29) for negative rescued embryos, Wilcoxon signed rank test on the differences from the positive embryos' mean was not statistically significant. D) Mean (standard deviation) cohort size was 10.56 (3.19) for positive cohorts versus 11.3 (3.64) for negative rescued embryos, Wilcoxon signed rank test on the differences from the positive embryos' mean was not statistically significant. E,F) Positive embryos that were erroneously classified as negative without cohort features and were correctly classified by a model that had access to cohort features. *N* = 62 rescued embryos. Distribution of the fraction of embryos within a cohort (not including the transferred embryo/s) to develop to a blastocyst (E) or cohort size (i.e., number of sibling embryos in a cohort) (F) compared across negative embryos versus positive embryos that were “rescued” by the cohort features, that is, correctly classified only by the classifier that had access to cohort information. E) Mean (standard deviation) fraction of sibling embryos within a cohort (not including the transferred embryo/s) reaching blastulation was 0.41 (0.25) for negative cohorts versus 0.56 (0.21) for positive rescued embryos, Wilcoxon signed rank test on the differences from the negative embryos' mean *p*‐value < 0.0001. F) Mean (standard deviation) cohort size was 9.9 (3.36) for negative cohorts versus 10.74 (3.17) for positive rescued embryos, Wilcoxon signed rank test on the differences from the negative embryos' mean *p*‐value < 0.01.

Finally, we evaluated the contribution of the cohort features to the classification of each of the transferred embryos. **Figure** **5**A shows the embryo classification scores by a model trained with morphology, morphokinetics, and oocyte age without (*x*‐axis) and with (*y*‐axis) cohort features. This model was selected for evaluation because it combined high performance and sufficient data volume. Each data point corresponds to an embryo and the color code indicates positive (green) and negative (red) embryos. Embryos above the *y* = *x* diagonal had higher classification scores when including the cohort features, reflecting a higher prediction for successful implantation. The inclusion of cohort features increased the classification scores of positive embryos (green data points above the *y* = *x* diagonal) and decreased the classification scores of negative embryos (red data points below the *y* = *x* diagonal), indicating that adding cohort features to the model improves model's discrimination for both positive and negative embryos (Figure 5B). To better understand how cohort features enhanced implantation prediction, we zoomed in on the subgroup of embryos that were “rescued” by the cohort features, that is, correctly classified only by the classifier that had access to cohort information. These included 15 non‐implanted embryos (negative) and 62 implanted (positive) “rescued” embryos from the test set of 388 (229 positive, 159 negative) embryos. We examined the two top‐ranked cohort features, fraction of sibling embryos reaching blastulation, and cohort size. We compared these features in cohorts of “rescued” embryos to all cohorts with the label corresponding to the “erroneous” prediction (by the model that did not have access to the cohort). For example, the two aforementioned cohort features of a positive embryo that was predicted as “negative” by a model that did not have access to cohort information and “rescued” by the cohort features, were compared to the corresponding features of all negative cohorts. This analysis should highlight whether these cohort features were correlated with the “rescued” prediction, and thus provide insight into what cohort features are used to improve the model's prediction. While not identifying an obvious pattern for rescued negative embryos (Figure 5C,D), we revealed in rescued positive embryos an elevation (relative to negative embryos) in the fraction of sibling embryos reaching blastulation and in the cohort size (Figure 5E,F). A similar pattern, although less reliable due to the lower number of rescued embryos (because of the smaller dataset), was observed for models that included the neural network's predictions (Figure S5, Supporting Information). These results suggest that these two cohort features were used by the model to correct negative‐to‐positive predictions but not positive‐to‐negative predictions.

## 3 Discussion

IVF is a suitable system for studying the effect of genotypic and environmental variation on phenotype. This is due to the availability of high‐content human embryo data that includes phenotypic information regarding multiple sibling embryos for each treatment cycle, who share a common genetic background and similar external conditions. From the machine learning perspective, IVF is a fitting example for an application where vast unlabeled data, specifically from non‐transferred cohort siblings, can provide valuable information for a more accurate prediction of embryo phenotypic quality, that is, implantation potential. These biological and machine learning concepts converge to a common theme where the uncertainty in the transferred embryo features, due to either inconsistency in annotations, features that were not explicitly measured, or label ambiguity, can be reduced by information encapsulated in the correlated cohort embryos. We believe that this is achieved by noise reduction with multiple correlated instances.

We established that embryos from the same cohort were more phenotypically similar than embryos from different cohorts (Figure 1), demonstrated that siblings of successfully implanted (positive) embryos were of higher phenotypic quality in relation to siblings of negative embryos (Figure 2), and demonstrated that cohort features contribute to machine learning‐based implantation prediction (Figure 3). The latter was achieved by extracting a new set of features from unlabeled siblings within the cohort, incorporating them with different feature sets, and comparing the classifier's performance in implantation prediction without versus with cohort features. Even though each individual cohort feature had only a marginal effect (Figure 2), the machine learning‐driven integration of all cohort features led to a consistent improvement in implantation prediction, regardless of the embryo‐focused model (Figure 3). These results suggest a general concept where the transferred embryo's siblings encapsulate discriminative information that is complementary to the information encoded in the transferred embryo. Thus, cohort features are likely to contribute to any embryo‐derived features.

Since the siblings' data are routinely collected in the clinic, incorporating cohort features in AI‐driven embryo implantation prediction can have direct translational implications in the clinic. The clinical significance of incorporating cohort features into our models was an AUC increase ranging between 0.04 and 0.1. Here, we show that such improvements can be achieved with no additional cost or effort by using the existing unlabeled embryo data with direct implications in clinical transparency and improving models for funding IVF treatments according to success probabilities, rather than a binary outcome. For example, in the best‐performing model, the cohort information improved the AUC from 0.727 to 0.8 (Figure 3G, visualized in Figure 5A,B).

We did not have access to Preimplantation Genetic Testing for Aneuploidy (PGT‐A) data in this study. Differences in embryo ploidy types (aneuploidy/euploidy) may introduce noise to the genotype that may alter the corresponding phenotype. Yet, we still show strong statistical relationships between sibling embryos, as well as strong associations between transferred embryos and their corresponding positive cohorts, even in the presence of this possible additional genotypic noise. This strengthens our stance regarding the strong genotypic‐phenotypic association between sibling embryos, and the benefit of using available unlabeled data of sibling embryos.

Previous studies correlated cohort‐based properties to implantation outcomes. For example, demonstrating improved outcomes for embryos selected from cohorts with more than five embryos,^[^ ^29^ ^]^ or from day 3 cohorts where at least one sibling embryo achieved blastulation after extended culture.^[^ ^32^, ^34^ ^]^ Other studies incorporated specifically designed cohort‐based features to machine learning models, specifically the cohort size^[^ ^28^, ^31^ ^]^ and the number of developed embryos,^[^ ^38^ ^]^ or even incorporated multiple cohort‐related features to show that the cohort alone contains discriminative information.^[^ ^30^ ^]^ We performed a comprehensive analysis that systematically assessed the contribution of the incorporation of cohort properties to existing models for the prediction of implantation outcomes. We did not decide on specific thresholds, rather we provided all the “raw” features to the AI machinery to automatically determine and weigh which and what combination was most discriminative. This unbiased approach allowed us to reveal how discriminative each cohort property is in the context of a given model (Figure 4) and which cohort properties were critical to “rescue” embryos that were incorrectly classified without cohort properties (Figure 5). Importantly, most of the previous studies mentioned above‐assessed embryos that were transferred at day 3 from fertilization. At this early stage of embryo development, the uncertainty of the implantation potential of a single embryo is higher,^[^ ^43^, ^44^ ^]^ and thus, additional correlated measurements in cohort features are expected to provide a more discriminative signal. This is especially relevant when sibling embryos are kept in extended blastocyst culture and their blastulation outcomes are known. Our results relate to blastocysts, that is, day 5 embryos, when the uncertainty is lower. Still, we were able to establish that the cohort information contributes discriminative power beyond the transferred embryo features.

We found that cohort features “rescued” fourfold more positive (*N* = 62) versus negative (*N* = 15) embryos (Figure 5). One explanation for this asymmetry could be due to the ambiguity in the negative labels. While positive embryos are inherently of high implantation quality, negative embryos fail implantation because of either low‐quality embryos and/or poor endometrial or uterine factors. This creates uncertainty in the ground truth labels of non‐implanted embryos, or “label ambiguity.”^[^ ^23^ ^]^ Thus, it could be easier to “rescue” a positive embryo that was mistakenly classified as “negative” with a quality cohort, in comparison to a negative embryo that could be of high implantation potential (also encoded in its cohort features).

In this study, digital embryo images were manually annotated for morphology (based on the Gardner embryo scoring system) and key morphokinetic events using the time‐lapse information. Manual annotation is throughput‐limiting. Automated tools for morphological evaluation^[^ ^25^, ^26^ ^]^ and detection of morphokinetic events^[^ ^23^, ^45^ ^]^ are quickly reaching human‐level performance^[^ ^46^ ^]^ and have the advantage of avoiding intra‐ and inter‐annotator bias potentially replacing the need for manual annotations in the near future.

In the context of machine learning, this is an example of semi‐supervised learning, where a small fraction of labeled (transferred embryos) and vast unlabeled observations (non‐transferred cohort siblings) are used together to improve the learned model's performance toward the task of predicting implantation potential. While the underlying assumption in semi‐supervised learning is that the observations are unrelated, here we characterize another type of semi‐supervised learning, where the unlabeled observations are associated with the labeled observations, and suggest a way to use information from these unlabeled observations for improving the prediction power of the supervised models.

## 4 Experimental Section

### Data Collection and Ethics

The data included in this analysis were retrospectively collected from IVF cycles conducted at a single center between March 2010 and December 2018. Historical images of blastocyst‐stage embryos and metadata were provided by AiVF. All procedures and protocols were approved by an Institutional Review Board for secondary research use (IRB reference number HMO‐006‐20). All IVF cycles were either autologous, with the patient's own oocytes (*n* = 1013, ≈63%) or with donor oocytes (*n* = 592, ≈37%), including 1328 (≈64%) autologous embryos and 761 (≈36%) donor embryos, ages 18–51 years old (Figure S6, Supporting Information). In cases of oocyte donation, donor age was considered as the “oocyte age.”

Fertilization was determined by the presence of two pronuclei (2PN) 16–18 h after insemination. All zygotes were placed inside the EmbryoScope time‐lapse incubator system (Vitrolife, Denmark) and incubated using sequential media protocol until the blastocyst stage. Image acquisition using the EmbryoScope imaging software occurred every 15–20 min. For every embryo, seven layers of *z*‐stack images 15 µm apart were acquired at each time point, where time 0 was defined as the fertilization time. A total of 16 194 oocytes from 1605 IVF cycles were recorded. All cycles included at least one fresh single embryo transfer at the blastocyst stage with a known implantation outcome (implanted/not implanted). Out of 2089 embryos with known implantation outcomes, 1176 were successfully implanted (positive embryos) and 913 failed to implant (negative embryos).

### Annotation of Embryo Clinical Quality

Following in vitro incubation, the number of embryos from the cohort transferred was either one or two. In cycles where two embryos were transferred, cases where both embryos either successfully implanted or both failed implantation were included. Implantation following embryo transfer was determined by ultrasound scanning for gestational sac after ≈7 weeks of pregnancy. Positive embryos (and their corresponding positive cohorts) were defined when the number of gestational sacs matched the number of transferred embryos. Negative embryos (and their corresponding negative cohorts) were defined when no gestational sac following embryo transfer was observed.

### Embryo Morphological Feature Representation

Embryos were morphologically annotated at the blastocyst stage (day 5 of embryonic development) according to the Gardner scoring scheme by an onsite trained embryologist ≈120 h post insemination.^[^ ^8^, ^37^ ^]^ The time of blastulation was determined by time‐lapse monitoring of embryo development. Specifically, every embryo was assigned a three‐part alphanumeric quality score based on its expansion status (“blastocyst expansion”, ranked 1–6), the morphology of the “ICM,” (ranked A–D), and morphology of the t“TE” (ranked A–D) (Figure 1H). Embryos with missing Gardner annotations were excluded from the analysis. Out of the 2089 embryos, 1936 had Gardner annotations, where 1141 were annotated as positive and 795 as negative. Embryos' morphological variables were computationally represented via one‐hot encoding, that is, a feature vector of size 6 + 4 + 4 = 14, representing the Gardner scores.

### Embryo Morphokinetic Feature Representation

Time‐lapse images of embryo development were viewed by an onsite trained embryologist and seven key morphokinetic events were manually annotated in accordance with published consensus criteria:^[^ ^47^ ^]^ Time of fertilization (t0), cell division to the 2,3,4, and 8‐cell stage (t2, t3, t4, t8), compaction of the morula (tM), and time of blastulation (tSB). Missing morphokinetic event annotations, either due to manual human error or the presence of unfocused, blurred, or missing images, were at levels of 5–10%, except for tM and tSB with 20% missing annotations, which was a limitation of the dataset. These missing annotations were completed according to the following rules, as determined by domain experts: t2 = time of pronuclei disappearance (tPNf – time when both pronuclei disappear, independently annotated, see below) + 2 h; t3 = t4 − 1 h; t4 = t3 + 1 h; t8 = t7 + 3 h; tM = tSB − 6 h. After manually completing the missing annotations, embryos with a further single missing value were determined by first finding the five most similar embryos based on the remaining available annotated morphokinetic features and then using their mean value of the missing features. Overall, the morphokinetic feature vector was of size 11 and included the (five) time intervals between every consecutive developmental stage and the (six) overall time points for each developmental stage from the time of fertilization. Importantly, the dataset also included manual annotations beyond the morphokinetic events listed above (e.g., tPNf, see above). These events were not included in the morphokinetic feature representation used in this analysis because many embryos in the dataset did not had these morphokinetic events annotated.

### Similarity between Timing of Morphokinetic Events

The similarity between the timings of morphokinetic events and between the timings of consecutive morphokinetic events between embryo pairs within the same cohort (siblings) and in different cohorts (non‐siblings) was compared. For each morphokinetic event timing and consecutive morphokinetic events timing, the similarity of all sibling and non‐sibling pairs was compared. A similarity measure that encoded the full morphokinetic profile was calculated as the Euclidean distance between normalized vectors that included the timing of all the morphokinetic events and the timing of all the consecutive morphokinetic events.

To compare morphokinetic similarities between sibling and non‐sibling embryo pairs with similar morphological properties, all embryo triplets that share the same Gardner scores were evaluated, where two embryos were siblings and the third embryo was from a different cohort.

For each triplet, the similarity in morphokinetics between the siblings versus the non‐siblings pairs was evaluated.

### Cohort Morphology‐Based Feature Representation

A cohort contained multiple embryos from the same couples in the same IVF treatment cycle. 17 cohort features were extracted from all cohort siblings, excluding the transferred embryo. These included the cohort size (the only feature that included the transferred embryo, the fraction of sibling embryos reaching blastulation (Figure 2B), the fraction of sibling embryos that hatched, a 13D vector encoding the fraction of siblings for each Gardner score (5 + 4 + 4 features).

### Automated Deep Learning‐Based Embryo Implantation Prediction

Access to historical images of 772 transferred blastocyst‐stage embryos from 638 cohorts and associated known implantation outcomes was there. Of these, 482 embryos successfully implanted (positive) and 290 failed to implant (negative).

### Images Preprocessing

The analysis focused on the blastocyst's last time frame prior to hatching and the center *z*‐stack image.

The raw frames were preprocessed in order to segment the embryo region from the surrounding well and background. This allowed to train more complex models on a large training set in a reasonable time while keeping the inherent spatial resolution features of the embryo. To localize and segment the embryo, the following pipeline was developed. First, a mask‐RCNN^[^ ^48^ ^]^ was trained to identify a bounding box around each embryo using 800 manually annotated images. Second, a Hough‐transform^[^ ^49^ ^]^ was applied to center the embryo in the image by detecting a circular object within the bounding box. Third, a U‐NET model was trained based on 500 manually validated outputs from the previous step to provide the embryo segmentation mask.^[^ ^50^ ^]^ The U‐NET architecture consisted of four convolutional layers for the encoder (downsampling)/decoder (upsampling) with 32, 64, 128, and 256 filters correspondingly. Each layer included batch normalization and relu activation function with maxpooling for the encoder and upsampling for the decoder. Finally, the image was further resized to 64 × 64. The trained pipeline architecture is presented in Figure S7, Supporting Information.

### Image Classification Model

A pretrained VGG16^[^ ^41^ ^]^ architecture was used as a backbone followed by a flattening layer, a fully connected 16‐node dense layer, and a dense single‐node layer with sigmoid activation. The full model was retrained (no freezing of weights) using binary cross entropy loss and the Adam optimizer with a learning rate of 0.00001 to output a confidence score in the range of 0–1 for predicting implantation probability.

The model was trained for 100 epochs with a batch size of 32. For each batch, image augmentation (brightness, flipping, rotation, and Gaussian noise) was performed prior to fitting the model to reduce overfitting. The model was trained with tenfold cross‐validation allowing all the available data points to run at inference and output a confidence score.

### Evaluation of Machine Learning Models Without Versus With Cohort Features

Models were trained with morphology features and/or morphokinetic features and/or the oocyte age. Each feature representation was used to train and then evaluate the performance of two models: without including cohort features (i.e., using only the transferred embryo information) versus including cohort features. The deep learning model was used as a feature extractor, where the model confidence score was one feature, and these features were used together with other features (e.g., morphokinetics, oocyte age) to train and then evaluate the performance of models that had versus those did not have access to cohort features. Models based on the same features without or with cohort features were compared to evaluate the contribution of cohort features to the overall prediction. The data was partitioned to train (80%) and test (20%) sets maintaining a similar ratio of positive (i.e., successful implantation) and negative (failed implantation) cohorts in both sets. An XGBoost binary classification model was trained for each feature set.^[^ ^51^ ^]^ When included, the confidence score of the deep learning model was considered as a single feature. ROC curve (i.e., true positive rate (TPR, sensitivity) versus false positive rate (FPR, 1‐specificity)) and its AUC were used to visually and quantitatively compare the performance of model pairs without versus with cohort features. Hyper‐parameter tuning was performed independently for each XGBoost classifier using sklearn's GridSearchCV.^[^ ^52^ ^]^ The parameters that were optimized were: 1) Number of gradient‐boosted trees, 2) minimum loss reduction required to make a further partition on a leaf node of the tree, 3) maximum tree depth for base learners, 4) subsample ratio of the training instance, 5) subsample ratio of columns when constructing each tree, and 6) minimum sum of instance weight (hessian) needed in a child.

Due to the lower volume of annotated raw image data, feature selection was applied, independently for each model, with the extremely Randomized Trees Classifier (ExtraTrees) classifier (an updated version of the random forest)^[^ ^53^ ^]^ to reduce the number of features to ten (unless the number of features was already lower).

### Feature Importance Analysis

For interpretability purposes, the feature importance method with SHAP was applied.^[^ ^42^ ^]^ SHAP computed the contribution of each feature to each individual prediction. Embryo morphological categorical parameters were transformed into ordinal variables for the SHAP analysis.

### Subgroup Analysis of Autologous Versus Donor Cycles

To assess whether the relations between siblings and non‐siblings change between autologous and donor cycles, a subgroup comparative analysis was performed. This analysis showed marginal differences in the cohort contribution to the prediction and in the ranking of specific cohort properties' importance. This analysis validated the inclusion of cohort information to enhance implantation prediction for both autologous and donor cohorts (Figure S8, Supporting Information). These results imply that autologous and donor cycles can be analyzed together to assess the contribution of cohort siblings to implantation prediction.

## Conflict of Interest

O.R., M.T.S., R.M., D.G., and D.S.S. are employees at AIVF LTD. M.M. is a paid advisor for AIVF Ltd. The remaining authors declare no conflict of interest.

## Author Contributions

N.T. and O.R. contributed equally to this work. A.Z. conceived the study. N.T. and O.R. developed analytic tools, and analyzed the data. N.T., O.R., and A.Z. interpreted the data and drafted the manuscript. M.T.S., R.M., M.M., D.G., and D.S.S. provided clinical input for the presentation of the manuscript, and reviewed and revised the manuscript. A.Z. mentored N.T. and O.R. All authors edited the manuscript and approved its content.

## Supporting information

Supporting Information

## Data Availability

The data that support the findings of this study are available from AIVF LTD. Restrictions apply to the availability of these data, which were used under license for this study. Data are available from the authors with the permission of AIVF LTD.
