# Supplementary material for: Using Unlabeled Information of Embryo Siblings from the Same Cohort Cycle to Enhance In Vitro Fertilization Implantation Prediction
Source: Adv Sci (Weinh). 2023 Jul 28;10(27):2207711. doi: 10.1002/advs.202207711 (PMC10520665; doi:10.1002/advs.202207711)
Supplement: Supplementary file 1 — Supporting Information [file ADVS-10-2207711-s001.pdf]

ADVANCED SCIENCE
Open Access

Supporting Information

for *Adv. Sci.*, DOI 10.1002/advs.202207711

Using Unlabeled Information of Embryo Siblings from the Same Cohort Cycle to Enhance In Vitro Fertilization Implantation Prediction

*Noam Tzukerman, Oded Rotem, Maya Tsarfati Shapiro, Ron Maor, Marcos Meseguer, Daniella Gilboa, Daniel S. Seidman and Assaf Zaritsky**

Supplementary Tables:

	# embryos (# test set)	# implanted embryos (# test set)	# non-implanted embryos (# test set)	# cohorts	# positive cohorts	# negative cohorts	# siblings
**Morphokinetics annotated**	2,089 (418)	1,176 (235)	913 (183)	1,605 (396)	928 (227)	677 (169)	14,105
**Morphology annotated**	1,936 (388)	1,141 (229)	795 (159)	1,513 (367)	908 (215)	605 (152)	13,493
**Image data**	772 (155)	482 (97)	290 (58)	638 (148)	404 (94)	234 (54)	5,990

Table S1: Number of transferred blastocysts, number of implanted blastocysts (positive embryos) and non-implanted blastocysts (negative embryos), number of cohorts, number of positive and negative cohorts and number of cohort's siblings for each dataset. The number of embryos used in test are marked in brackets.

Supplementary Figures:

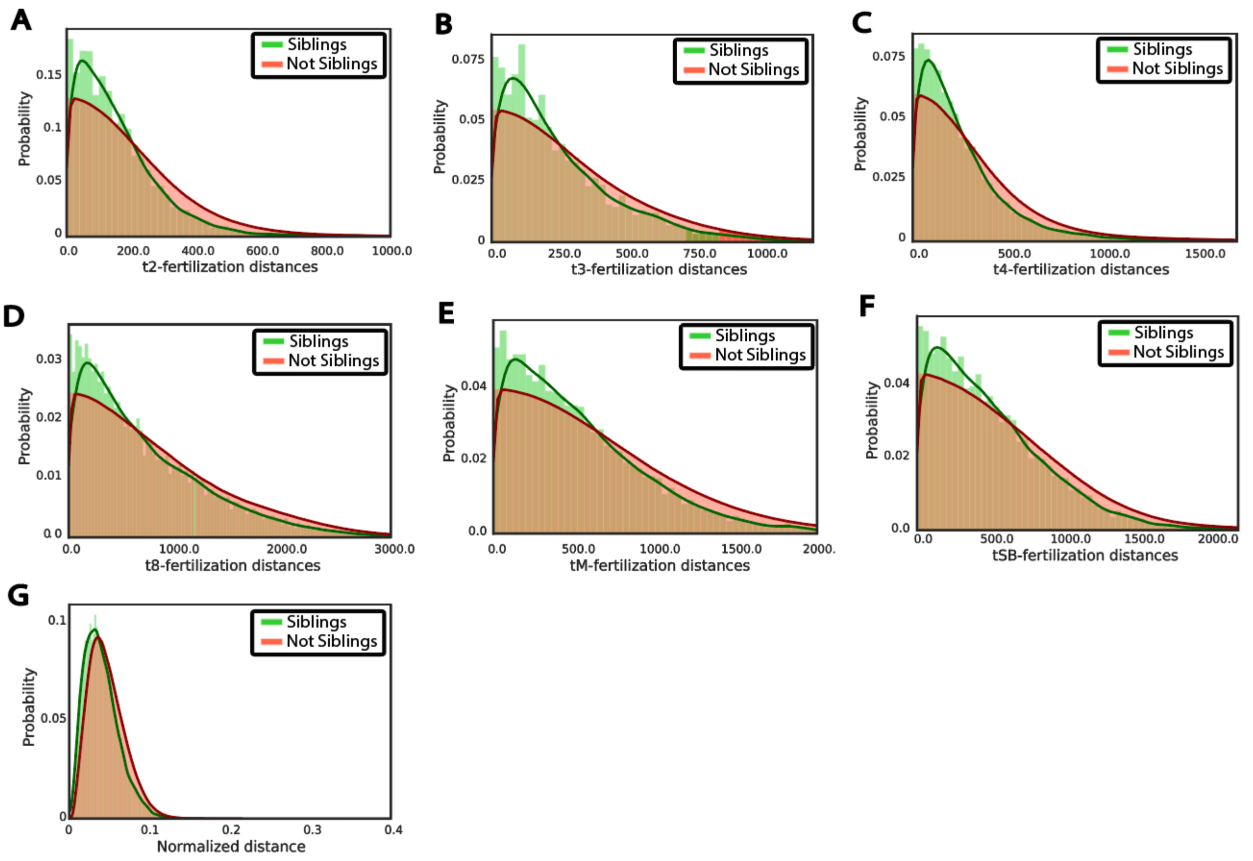

Figure S1. Sibling embryos from the same cohort are more similar than non-siblings. (**A-F**) Distribution of the difference in time intervals, in minutes (A-F) or normalized distance (G), between fertilization and each morphokinetic event compared across siblings versus non-siblings embryo pairs. morphokinetic features: cell division to the 2, 3, 4 and 8-cell stage (t2, t3, t4, t8), the compaction of the morula - a day-3 development stage (tM) and the start of blastulation (tSB) - a day-5 development stage. N embryos = 16194. N cohorts = 1605. N positive cohorts = 928, N negative cohorts = 677. (**A**) Mean (standard deviation) of distances between fertilization-t2 intervals was 158.56 (150.59) for sibling embryos versus 217.62 (218.51) for non-sibling embryos, Mann-Whitney-U signed rank test p-value < 0.0001. (**B**) Mean (standard deviation) of distances between fertilization-t3 intervals was 258.21 (238.36) for sibling embryos versus 327.68 (290.56) for non-sibling embryos, Mann-Whitney-U signed rank test p-value < 0.0001. (**C**) Mean (standard deviation) of distances between fertilization-t4 intervals was 261.45 (268.56) for sibling embryos versus 333.01 (314.93) for non-sibling embryos, Mann-Whitney-U signed rank test p-value < 0.0001. (**D**) Mean (standard deviation) of distances between fertilization-t8 intervals was 700.46 (595.04) for sibling embryos versus 836.65 (666.99) for non-sibling embryos, Mann-Whitney-U signed rank test p-value < 0.0001. (**E**) Mean (standard deviation) of distances between fertilization-tM intervals was 537.38 (443.54) for sibling embryos versus 655.45 (515.67) for non-sibling embryos, Mann-Whitney-U signed rank test p-value < 0.0001. (**F**) Mean (standard deviation) of distances between fertilization-tSB intervals was 499.29 (397.18) for sibling embryos versus 596.34 (462.68) for non-sibling embryos, Mann-Whitney-U signed rank test p-value < 0.0001. (**G**) Mean (standard deviation) of normalized distances between all morphokinetic features time intervals from fertilization was 0.04 (0.02) for sibling embryos versus 0.05 (0.02) for non-sibling embryos, Mann-Whitney-U signed rank test p-value < 0.0001.

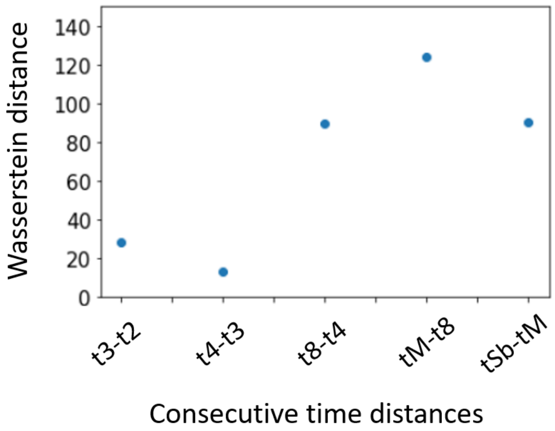

Figure S2: Differences between intra-cohort and inter-cohort morphokinetic events duration along the embryo development. The Wasserstein distance (aka "earth mover distance") between the distributions of siblings and non-siblings morphokinetic events durations (Figure 1B-F). Larger inter-intra differences are observed in later stages.

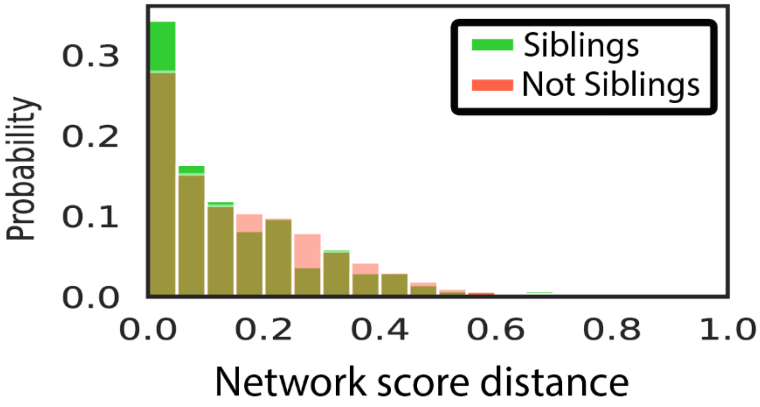

Figure S3: Transferred sibling embryos from the same cohort are more morphologically similar than transferred non-sibling embryos. Embryo morphology was encoded by the image-based deep learning network scores. Distributions of the Manhattan (L1) distance of network scores compared across transferred sibling pairs from the same cohort (N = 134 pairs) versus non-sibling transferred embryo pairs (N = 594,944). Mean (standard deviation) siblings distance was 0.14 (0.14) versus 0.17 (0.14) for non-sibling embryos, Mann-Whitney-U signed rank test p-value < 0.055.

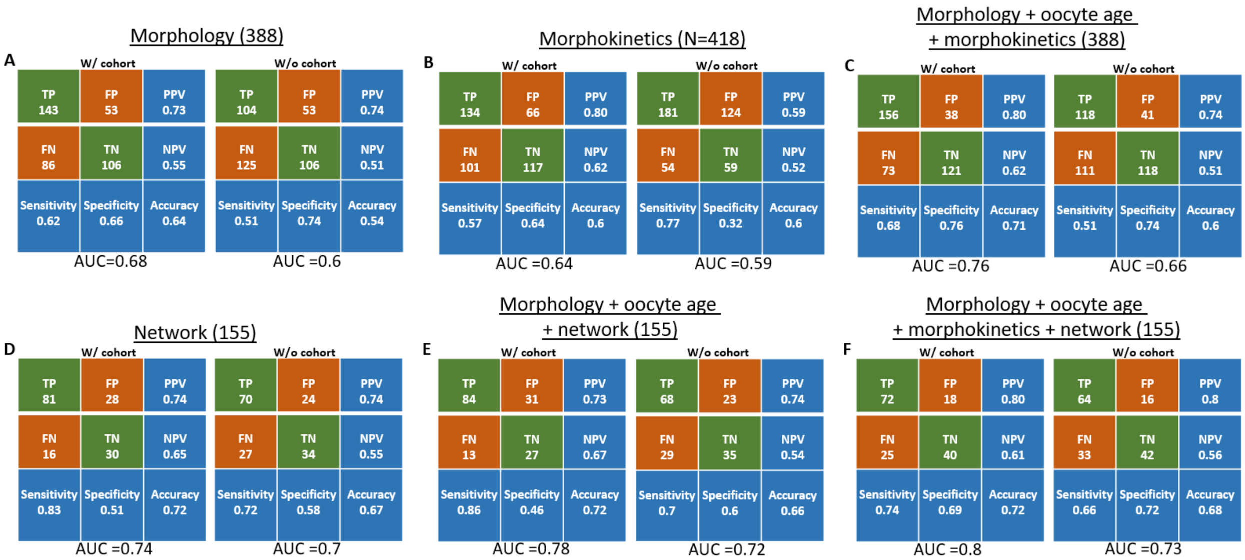

Figure S4: Implantation prediction performance (confusion matrix) comparison for all models with cohort features (left) versus without. (**A**) Morphology. (**B**) Morphokinetics. (**C**) Morphology, oocyte age and morphokinetics. (**D**) Network. (**E**) Morphology, oocyte age and network. (**F**) Morphology, oocyte age, morphokinetics and network.

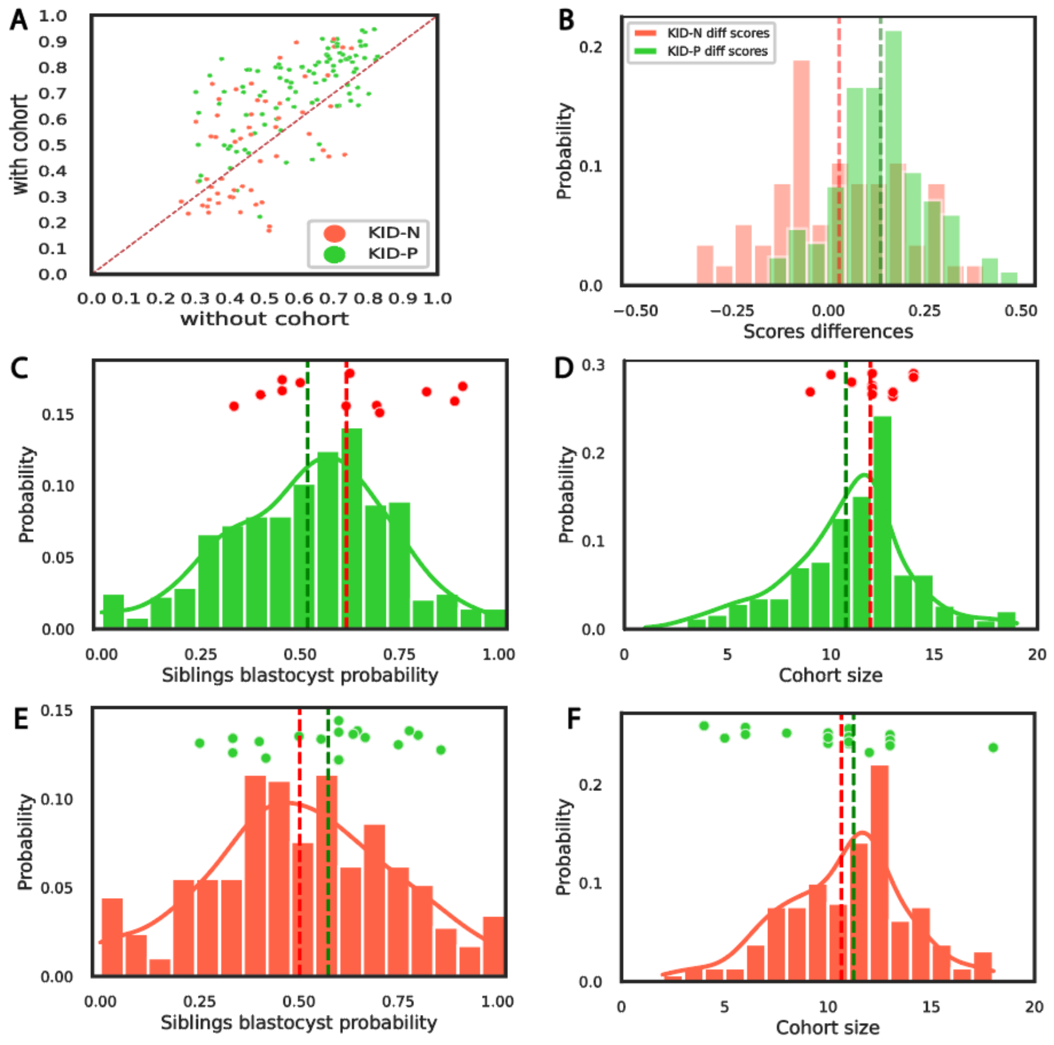

Figure S5: Analysis of cohort properties that "rescued" erroneous prediction. N transferred blastocysts = 772 from which 482 were positive and 290 were negative embryos. The results refer to a model trained with deep convolutional neural network, morphology, morphokinetics and oocyte age without and with cohort features. **(A)** Embryos matched classification scores by the two models: without (x-axis) and with (y-axis) cohort features. **(B)** Distribution of the difference in the embryos matched classification scores: with - without cohort features. Mean (standard deviation) difference for positive cohorts was 0.13 (0.11) (Wilcoxon rank-sum test p-value < 0.0001) versus 0.01 (0.17) (Wilcoxon rank-sum test was not statistically significant) for negative cohorts. **(C-F)** Distribution of fraction of blastocysts siblings (C,E) or cohort size (D,F) for positive (green, C-D) or negative (red, E-F) embryos. Each of the data points above the distribution indicate an embryo that was "rescued" with the cohort feature, i.e., classified erroneously by a model trained without and corrected with a model trained with cohort features. **(C-D)** Negative embryos that were erroneously classified as positive without cohort features and were correctly classified by a model that had access to cohort features. N = 12 rescued embryos.(**C**) Mean (standard deviation) fraction of sibling embryos within a cohort (not including the transferred embryo/s) reaching blastulation was 0.51 (0.2) for positive cohorts versus 0.61 (0.19) for negative rescued embryos, Wilcoxon signed rank test on the differences from positive embryos mean found no statistical

significance. (**D**) Mean (standard deviation) cohort size was 10.56 (3.19) for positive cohorts versus 11.91 (3.64) for negative rescued embryos, Wilcoxon signed rank test on the differences from positive embryos mean found no statistical significance. **(E-F)** Positive embryos that were erroneously classified as negative without cohort features and were correctly classified by a model that had access to cohort features. N = 18 rescued embryos. Distribution of the fraction of embryos within a cohort (not including the transferred embryo/s) to develop to a blastocyst (E) or cohort size (i.e., number of sibling embryos in a cohort) (F) compared across negative embryos versus positive embryos that were "rescued" by the cohort features, i.e., correctly classified only by the classifier that had access to cohort information. (**E**) Mean (standard deviation) fraction of sibling embryos within a cohort (not including the transferred embryo/s) reaching blastulation was 0.5 (0.23) for negative cohorts versus 0.57 (0.17) for positive rescued embryos, Wilcoxon signed rank test on the differences from negative embryos mean found no statistical significance (p-value = 0.07). (**F**) Mean (standard deviation) cohort size was 10.64 (3.03) for negative cohorts versus 10.11 (3.39) for positive rescued embryos, Wilcoxon signed rank test on the differences from negative embryos mean found no statistical significance.

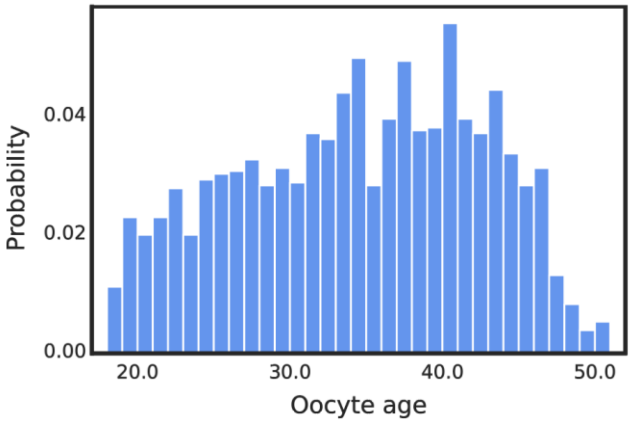

Figure S6: Distribution of oocyte age for all treatments. N= 2089. N cohorts = 1605. Mean (standard deviation) oocyte age was 33.99 (7.98).

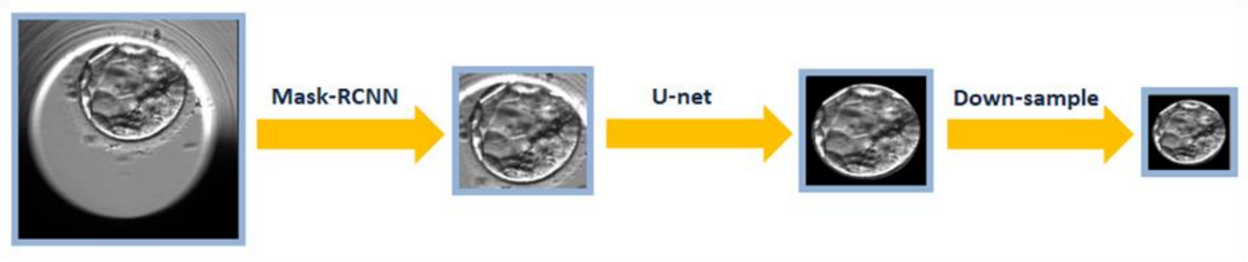

Figure S7: Oocyte segmentation pipeline (left-to-right). Mask-RCNN detects the embryo's bounding box. The cropped image is segmented by a U-NET network. Finally the image is down sampled to 64x64.

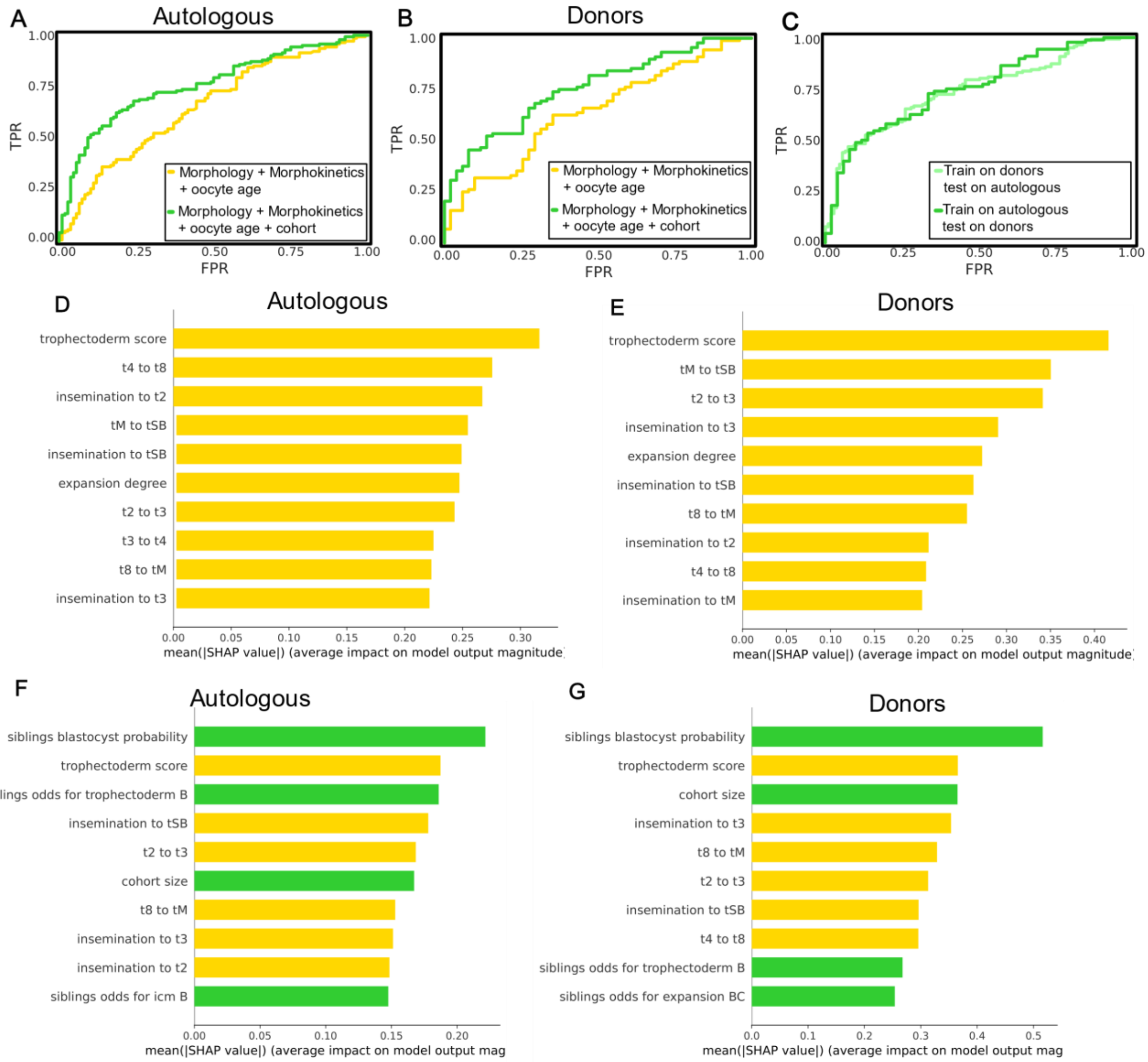

Figure S8: Subgroup analysis of N = 1,936 embryos: autologous (N = 1,242) versus donor (N = 694) embryos using the models trained with morphology, morphokinetics and oocyte age, with or without siblings features. Models trained and tested for each subgroup independently. **(A-C)** Implantation prediction of autologous (A) versus egg donation (B) cycles. Performance was measured with receiver operating characteristic (ROC) area under the curve (AUC). (**A-B**) Comparison of models trained with versus without sibling features. **(A)** Autologous: N embryos = 1,242 (N test = 249). N positive embryos = 702, N negative embryos = 540 (108). AUC: 0.65 versus 0.76, respectively, p-value < 0.001. **(B)** Donor: N embryos = 694 (139). N positive embryos = 439 (88), N negative embryos = 255 (51). AUC: 0.63 versus 0.75, respectively, p-value < 0.001. **(C)** Cross subgroup evaluation. Bright green - Model trained on donors data (B) evaluated on autologous data (A), dark green - vise-versa. AUC: 0.74 versus 0.75, respectively, p-value not significant. **(D-G)** Model explainability analysis. Features importance for the top ten features using Shapely Additive Explanations (ShAP). Autologous cycles without (D) or with (F) cohort features. Donor cycles without (E) or with (G) cohort features. Autologous and donor cycles were mostly consistent both in terms of cohort contribution and in terms of cohort features explainability.
